# Morphology and molecular phylogeny reveal two new species of *Ganoderma* (*Basidiomycota*, *Polyporales*) from China and Fiji

**DOI:** 10.3897/mycokeys.139.208287

**Published:** 2026-08-20

**Authors:** Xin-Yan Liu, Yan-Ling Liu, Zhan-Xi Lin, Dong-Mei Lin, Xing-Sheng Lin, Yong-Mei Cheng, Yi-Fei Sun, Bao-Kai Cui

**Affiliations:** 1 National Key Laboratory for Efficient Production of Forest Resources, School of Ecology and Nature Conservation, Beijing Forestry University, Beijing 100083, China National Engineering Research Center of Juncao Technology, Fujian Agriculture and Forestry University Fuzhou China; 2 National Engineering Research Center of Juncao Technology, Fujian Agriculture and Forestry University, Fuzhou 350002, China National Key Laboratory for Efficient Production of Forest Resources, School of Ecology and Nature Conservation, Beijing Forestry University Beijing China; 3 Shenzhen Baoding Industrial Co., Ltd., Shenzhen 518000, China Shenzhen Baoding Industrial Co., Ltd. Shenzhen China

**Keywords:** *
Ganodermataceae
*, macrofungi, novel species, phylogeny, taxonomy

## Abstract

*Ganoderma* is a group of macrofungi with significant economic and medicinal values. In this study, two new species, *Ganoderma ficuum* and *G. fijiense*, are described based on morphological characteristics and molecular phylogenetic analyses. *Ganoderma ficuum* is characterized by imbricate and highly umbonate pilei, with relatively coarse radial sulcations, circular to angular pores (6–8 per mm), ellipsoid and distinctly truncated basidiospores, and from Guangdong, China. *Ganoderma fijiense* is characterized by reddish brown to dark brown pilei, with usually obtuse margins, circular pores (5–7 per mm), narrowly ellipsoid basidiospores, with the endospore wall bearing densely spinulose ornamentation, and from Nadi, Fiji. In addition, multi-gene phylogenetic analyses showed that *G. fijiense* and *G. ficuum* formed two independent lineages with well supports based on the internal transcribed spacer (ITS), translation elongation factor 1-α gene (*TEF1-α*), and the second largest subunit of RNA polymerase II (*RPB2*) genes using Maximum Likelihood (ML) and Bayesian Inference (BI) methods. The morphological descriptions and illustrations for two new species are also provided.

## Introduction

*Ganoderma* belongs to the family *Ganodermataceae* in *Polyporales* with wide distribution in the world, including Asia, Europe, North America, South America, Africa, and Oceania, and most species mainly occur in tropical and subtropical regions (Luangharn et al. 2021; Wu et al. 2022; Hyde et al. 2024; Zhao et al. 2024, 2026). As important edible and medicinal fungi, *Ganoderma* has a long history of medicinal use for maintaining vitality and prolonging life, especially in Asian countries (Si et al. 2019; Wu et al. 2019; Dai et al. 2021; Bhunjun et al. 2024; Karunarathna et al. 2025). *Ganoderma* contains diverse bioactive constituents, such as polysaccharides, triterpenoids, and other microelements, with immunomodulation, antitumor, hypoglycemic, and other pharmacological properties (Wang et al. 2025). Moreover, as white-rot fungi, *Ganoderma* possesses the ability to efficiently degrade lignocellulose that plays an important role in nutrient cycling (Zhou et al. 2013). However, some species are also invasive pathogens of forest trees, capable of secreting lignin peroxidase, manganese peroxidase, and other enzymes to degrade wood structure, causing butt-rot and root-rot, etc. (D’Souza et al. 1999; Torres-Farradá et al. 2017; Yuan et al. 2023). For example, *Ganoderma boninense* Pat., *G. philippii* (Bres. & Henn. ex Sacc.) Bres., and *G. tsugae* Murrill etc. can infect rubber, oil palm, *Casuarina*, fir, larch, and other trees, causing serious economic losses to forest plantations and urban street trees (Dai et al. 2000; Rees et al. 2010; Qin et al. 2014; Jiang et al. 2021; Bharudin et al. 2022).

*Ganoderma* is the most species-rich genus in *Ganodermataceae*, established in 1881 with its type species *G. lucidum* P. Karst. (Karsten 1881; Sun et al. 2022; Mardones et al. 2023). The main characteristics of this genus are: basidiomata sessile to stipitate, with laccate or non-laccate surface, basidiospores with a double-walled structure, and the inner wall with columnar or ridge-like pigmented structures (Loyd et al. 2018; Mardones et al. 2023). Moncalvo and Ryvarden (1997) documented 148 species of *Ganoderma* based on morphological characters, yet more than half of these taxa were later defined as different phenotypic variations (Jargalmaa et al. 2017). With the development of molecular techniques, researchers have used multiple genes, such as ITS, nLSU, *TEF1-α* and *RPB2*, for species identification and genetic diversity analyses (Moncalvo et al. 1995; Smith and Sivasithamparam 2000), effectively compensating for the shortcomings of traditional morphological identification. Wang et al. (2012) constructed a relatively comprehensive phylogenetic framework for the genus *Ganoderma* based on ITS and nLSU sequences, preliminarily revealing that the genus is monophyletic. Subsequently, Zhou et al. (2015) further identified multiple evolutionary clades within *Ganoderma* through multilocus phylogenetic analyses combining ITS, *TEF1-α*, *RPB1*, and *RPB2*. Cabarroi-Hernández et al. (2019) employed both single-gene (ITS) and multilocus (ITS+*RPB2*+*TEF1-α*) approaches to analyze the *G. weberianum-resinaceum* lineage, demonstrating that multilocus phylogenetic analysis can effectively resolve the phylogenetic positions of complex groups.

*Ganoderma* has a wide host range, mainly growing on dead wood, stumps, or roots of various living trees (Pilotti 2005). In natural environments, they particularly favor oaks and chestnuts of *Fagaceae*, birches and alders of *Betulaceae*, as well as common broad-leaved trees such as maples and lindens (Terho et al. 2007). Meanwhile, plants of *Arecaceae* such as coconut, oil palm, and areca-nut are also important hosts of *Ganoderma* fungi (Thakur et al. 2025), including *G. boninense*, *G. zonatum* Murrill, *G. chalceum* (Cooke) Steyaert, *G. tornatum* (Pers.) Bres., *G. applanatum* (Pers.) Pat., *G. hochiminhense* Karunarathna et al. and *G. orbiforme* (Fr.) Ryvarden. Future efforts to explore *Ganoderma* resources should further broaden the range of host plants.

China is one of the important distribution areas of *Ganoderma*, where its vast territory, complex landforms, and multiple climatic zones ranging from tropical to cold-temperate can provide suitable environmental conditions for the growth of *Ganoderma* fungi (He et al. 2022; Cui et al. 2023). New species of *Ganoderma* have been continuously identified. By June 2026, 53 species of *Ganoderma* had been discovered in China, of which 52 have ITS sequences available for molecular identification (He et al. 2021, 2022, 2024; Sun et al. 2022; Yang et al. 2022; Cui et al. 2023; Wei et al. 2024; Tian et al. 2025; Xu et al. 2025). Pacific Island countries are located in the South Pacific region, covering more than ten independent countries and territories including Fiji, Kiribati, Samoa, etc. *G. chalceum*, *G. tornatum*, *G. weberianum* (Bres. & Henn. ex Sacc.) Steyaert, *G. philippii* and *G. pfeifferi* Bres. were found in Pacific Island countries (Smith and Sivasithamparam 2003; Pilotti et al. 2004). Both localities harbor a wealth of *Ganoderma* fungal resources that warrant further investigation.

In this study, 17 specimens collected from China, Fiji and other locations were identified through morphological and multi-locus phylogenetic analyses, which included two new species. This study once again enhances our understanding of the species diversity of the genus *Ganoderma*.

## Materials and methods

### Specimen collections

The 17 specimens used for sequencing in this study were collected in field surveys in China, South Africa, Fiji, and other localities from March 2019 to July 2025. Specimens were photographed in the field, then removed from the substrate as completely as possible, while recording the collection date, locality, host plant, collector, and altitude, and were assigned corresponding numbers (Rathnayaka et al. 2025). The tested specimens were deposited in the Fungal Herbarium of the Institute of Microbiology, Beijing Forestry University (BJFC, Beijing, China).

### Morphological studies

Macro-morphological descriptions were based on field records and preserved specimens, and micro-morphological data were obtained from sections of dried specimens. Observations and micro-photographs were taken using a Nikon Eclipse 80i microscope (Nikon Corporation, Tokyo, Japan). Dried specimens were cut into thin sections with a razor blade, placed on glass slides, mounted in 5% potassium hydroxide (KOH) solution, and then Melzer’s reagent was added to highlight tissue structures. The prepared slides were first examined under a 40× objective to locate target structures, then observed under a 100× oil-immersion objective. Micromorphological features observed included: spore color, size, presence of apiculus and truncation; types and diameters of context hyphae and tube hyphae, etc. Measurements were performed using ImageJ software. To reflect variation in basidiospore size, 5% of the data at each end of the measurement range were excluded, and the specific values are reported in parentheses (Sun et al. 2022; Dong et al. 2025). The following abbreviations are used: **IKI** = Melzer’s reagent, **IKI–** = neither amyloid nor dextrinoid, **KOH** = 5% potassium hydroxide, **CB** = Cotton Blue, **CB+** = cyanophilous, **L** = mean spore length (arithmetic average of all spores), **W** = mean spore width (arithmetic average of all spores), **Q** = variation in the L/W ratios between the specimens studied, **n(a/b)** = number of spores: (**a**) number of measurements per sample, (**b**) number of samples.

### DNA extraction, amplification and sequencing

The novel plant genome (CTAB) rapid plant genome extraction kit-DN15 (Aidlab Biotechnologies Co., Ltd., Beijing, China) was used (Xing et al. 2018; Sun et al. 2020; Yang et al. 2025). The PCR primers used in this study are shown in Table 1.

**Table 1.** Primers used in this study for PCR analyses.

**Locus**	**Primers**	**Primer sequences**	**References**
ITS	ITS4	TCCTCCGCTTATTGATATGC	White et al. 1990
	ITS5	GGAAGTAAAAGTCGTAACAAGG	
*TEF1-α*	TEF1-983F	GCYCCYGGHCAYCGTGAYTTYAT	Matheny et al. 2006
	TEF1-1567R	ACHGTRCCRATACCACCRATCTT	
*RPB2*	fRPB2-5F	GAYGAYMGWGATCAYTTYGG	Liu et al. 1999
	fRPB2-7cR	CCCATRGCTTGYTTRCCCAT	

The PCR amplification system (50 µL) was composed as follows: 2× EasyTaq® PCR SuperMix (25 µL), ddH_2_O (20 µL), forward primer (1.5 µL), reverse primer (1.5 µL), and template DNA (2 µL). The cycling program for ITS (650 bp) was: initial denaturation at 95 °C for 3 min; denaturation at 94 °C for 40 s, annealing at 54 °C for 45 s, and extension at 72 °C for 1 min (these three steps repeated for 35 cycles); final extension at 72 °C for 10 min, and hold at 4 °C. The cycling program for *TEF1-α* (650 bp) was the same as that for ITS, with an annealing temperature of 56 °C; the cycling program for *RPB2* (1200 bp) was: initial denaturation at 95 °C for 10 min; denaturation at 94 °C for 1 min, annealing at 56 °C for 1 min, and extension at 72 °C for 1.5 min (these three steps repeated for 39 cycles); final extension at 72 °C for 10 min, and hold at 4 °C. The PCR amplification products were sent to BGI Genomics Co., Ltd. for purification and sequencing, and the sequencing primers used were identical to the amplification primers.

### Sequence alignment and phylogenetic analyses

The newly generated sequences in this study have been deposited in GenBank. All sequences except those generated in this study were obtained from GenBank for phylogenetic analyses. *Haddowia macropora* JV 1908/46 was used as outgroup (Sun et al. 2022). Sequence alignment was performed with MAFFT v.7 (Katoh and Standley 2013), and the alignment was manually adjusted in BioEdit v.7.0.9 (Hall 1999). The aligned ITS, *TEF1-α*, and *RPB2* sequences were concatenated using Mesquite v.3.2. (Maddison and Maddison 2004). Phylogenetic tree construction was performed using Maximum Likelihood (ML) and Bayesian Inference (BI). Maximum Likelihood analyses were conducted using RAxML v.8.2.12 (Stamatakis 2014) with the GTR+I+G model, and branch support was assessed with 1,000 bootstrap replicates. The optimal substitution model for phylogenetic reconstruction was selected using jModelTest 2.1.10. (Darriba et al. 2012). Bayesian Inference was performed using MrBayes v.3.2 (Huelsenbeck 2012) via MCMC, with four parallel chains run for five million generations, sampling every 1,000 generations, and discarding the first 25% of trees as burn-in; the remaining trees were used to calculate Bayesian posterior probabilities (BPP). The phylogenetic trees were visualized using FigTree 1.4.3 (http://tree.bio.ed.ac.uk/software/figtree/). Font sizes and lines of the text in the phylogenetic tree were edited using Adobe Illustrator CS5. Branches with ML bootstrap support (ML-BS) ≥ 70% and BPP ≥ 0.95 were considered as significantly supported.

## Results

### Molecular phylogeny

A total of 363 *Ganoderma* sequences (137 ITS, 125 *TEF1-α*, and 101 *RPB2*) were collected, including 37 newly sequenced, and used to construct the phylogenetic tree. These sequences were derived from 132 specimens representing 67 *Ganoderma* species (including two new species). The phylogenetic tree constructed by Maximum Likelihood (ML) analyses had a topology similar to that of the tree constructed by Bayesian Inference (BI) analyses, with only minor differences in the positions of a few branches.

*Ganoderma ficuum* sp. nov. formed a sister clade to *G. weberianum*, *G. sichuanense* J.D. Zhao & X.Q. Zhang, *G. mexicanum* Pat. and *G. parvulum* Murrill. *Ganoderma fijiense* sp. nov. formed a distinct branch and clustered with *G. zonatum*, *G. cocoicola* B.K. Cui et al., *G. hochiminhense* and *G. boninense*.

The ITS sequences of *G. ficuum* (Cui 23393) and *G. fijiense* (202305FJ) were also subjected to BLAST searches against the GenBank database. For *G. ficuum*, the top ten hits showed high similarity with three *Ganoderma* species: 98.16% and 98.11% with *G. sichuanense* (ZD16072301, BMP214), 98.32% with *G. weberianum* (CSH38, GW-10, GW-11), and 98.55–98.10% with *G. subamboinense* (SG0122-3, Liu 787). For *G. fijiense*, the top ten hits showed 97.18–96.71% similarity with *G. boninense* (R.09C, R.06C). The differences between the sequences of the two new species and the existing sequences in the GenBank database were obvious (Table 2).

**Table 2.** Specimen information and GenBank accession numbers used in the phylogenetic analyses.

**Species**	**Specimens**	**Geographic origin**	**GenBank accession numbers**		
			**ITS**	***RPB2***	***TEF1-α***
*Ganoderma acontextum*	JV 0611/21G^T^	Guatemala	KF605667	MG367489	MG367538
*G. acontextum*	JV 1208/11J	Guatemala	KF605668	MG367490	MG367540
*G. adspersum*	Dai 13191	France	MG279153	MG367492	MG367541
*G. adspersum*	HSBU200894	Iran	MG279154	–	MG367542
*G. adspersum*	Cui 24149	Xizang, China	**PZ630499**	–	**PZ661913**
*G. alpinum*	Cui 18402	Xizang, China	MZ354910	–	**PZ661914**
*G. angustisporum*	Dai 19603	Sri Lanka	MZ354978	MZ245385	MZ221633
*G. angustisporum*	Dai 19649	Sri Lanka	**PZ630500**	**PZ661909**	**PZ661915**
*G. angustisporum*	Cui 18240	Malaysia	MZ354979	MZ245386	MZ221634
*G. applanatum*	Cui 14062	Jilin, China	MZ354913	MZ358846	MZ221635
*G. applanatum*	Cui 14070	Jilin, China	MZ354914	MZ245387	MZ221636
*G. artocarpicola*	HL 188	Yunnan, China	ON994240	OP508427	OP508441
*G. artocarpicola*	HL 173^T^	Yunnan, China	ON994239	OP508428	OP508442
*G. australe*	Cui 17254	Guangdong, China	MZ354966	MZ245406	MZ221660
*G. australe*	Cui 24291	Hunan, China	**PZ630501**	–	**PZ661916**
*G. baisuzhenii*	FHMU7350	Hainan, China	PP663110	PP785031	PV066219
*G. boninense*	WD2085	Japan	KJ143906	KJ143965	KJ143925
*G. boninense*	WD2028	Japan	KJ143905	KJ143964	KJ143924
*G. brownii*	JV 1105/9J	United States	MG279159	MG367494	MG367547
*G. brownii*	JV 0709/109	United States	KF605662	MG367495	MG367548
*G. bubalinomarginatum*	Dai 20074	Guangxi, China	MZ354926	MZ245389	MZ221638
*G. bubalinomarginatum*	Dai 20075^T^	Guangxi, China	MZ354927	MZ245388	MZ221637
*G. casuarinicola*	Dai 19470	Sri Lanka	MZ354996	MZ245392	MZ221642
*G. casuarinicola*	Dai 16336^T^	Guangdong, China	MG279173	MG367508	MG367565
*G. cocoicola*	Cui 16791^T^	Australia	MZ354984	MZ245393	MZ221643
*G. cocoicola*	Cui 16792	Australia	MZ354985	MZ245394	MZ221644
*G. curtisii*	CBS 100131	United States	JQ781848	KJ143966	KJ143926
*G. curtisii*	CBS 100132	United States	JQ520164	KJ143967	KJ143927
*G. dianzhongense*	L4969	Yunnan, China	MW750240	MZ467044	MW838996
*G. dianzhongense*	L4331^T^	Yunnan, China	MW750237	MZ467043	MW838993
*G. ecuadorense*	URM 89449	Ecuador	MK119828	MK121535	MK121577
*G. ecuadorense*	URM 89441	Ecuador	MK119827	MK121534	MK121576
*G. eickeri*	CMW 49692^T^	South Africa	MH571690	–	MH567287
*G. eickeri*	Dai 12595	South Africa	**PZ630502**	–	**PZ661917**
*G. ellipsoideum*	CMW 14080966^T^ 14080966(TYPE)	Hainan, China	MH106867	–	–
*G. ellipsoideum*	Cui 23479	Hainan, China	**PZ630503**	–	**PZ661918**
*G. ellipsoideum*	Dai 19545	Sri Lanka	**PZ630504**	**PZ661910**	**PZ661919**
*G. enigmaticum*	Dai 15970	Africa	KU572486	MG367513	KU572496
*G. enigmaticum*	Dai 15971	Africa	KU572487	MG367514	KU572497
***G. ficuum***	**Cui 23393^T^**	Guangdong, China	**PZ630505**	–	**PZ661920**
***G. ficuum***	**Cui 23385**	Guangdong, China	**PZ630506**	–	**PZ661921**
***G. ficuum***	**Cui 23386**	Guangdong, China	**PZ630507**	–	**PZ661922**
***G. fijiense***	**202305FJ^T^**	Fiji	**PZ630508**	**PZ661911**	**PZ661923**
*G. flexipes*	Cui 13841	Hainan, China	MZ354923	MZ245401	MZ221655
*G. flexipes*	Cui 13863	Hainan, China	MZ354924	MZ245402	MZ221656
*G. gibbosum*	Cui 23424	Zhejiang, China	**PZ630509**	–	**PZ661924**
*G. gibbosum*	Cui 24349	Shanxi, China	**PZ630510**	–	**PZ661925**
*G. guangxiense*	Cui 14453^T^	Guangxi, China	MZ354939	MZ245407	MZ221661
*G. guangxiense*	Cui 14454	Guangxi, China	MZ354941	MZ245408	MZ221662
*G. guixiense*	GXU3457^T^	Guangxi, China	OQ788244	PP187389	–
*G. guixiense*	GXU3709	Guangxi, China	OR271986	–	–
*G. hochiminhense*	MFLU 19-2224^T^	Yunnan, China	MN398324	–	MN423176
*G. hochiminhense*	MFLU 19-2225	Yunnan, China	MN398325	–	MN423177
*G. hoehnelianum*	Dai 11995	Yunnan, China	KU219988	MG367497	MG367550
*G. hoehnelianum*	Cui 13982	Guangxi, China	MG279178	MG367515	MG367570
*G. knysnamense*	CMW 47755^T^	South Africa	MH571681	–	MH567261
*G. knysnamense*	CMW 49688	Africa	MH571683	–	MH567266
*G. leucocontextum*	Dai 15601	Xizang, China	KU572485	MG367516	KU572495
*G. leucocontextum*	GDGM 40200^T^	Xizang, China	KF011548	–	–
*G. lingzhi*	Dai 12574	Liaoning, China	KJ143908	JX029981	JX029977
*G. lingzhi*	Cui 9166	Shandong, China	KJ143907	JX029978	JX029974
*G. lobatum*	JV100831	United States	KF605671	MG367499	MG367553
*G. lobatum*	JV100832	United States	KF605670	MG367500	MG367554
*G. lucidum*	Cui 14405	Sichuan, China	MG279182	MG367520	MG367574
*G. lucidum*	CCBAS707	Europe	MG706231	MG837805	MG837846
*G. martinicense*	UMNTN1	United States	MG654178	MG754860	MG754738
*G. martinicense*	He 2240	United States	MG279163	MG367503	MG367557
*G. meredithiae*	UMNFL50	United States	MG654103	MG754862	MG754735
*G. meredithiae*	CBS 271.88^T^	United States	NR_164435	–	–
*G. mexicanum*	MUCL 55832	Martinique	MK531815	MK531839	MK531829
*G. mexicanum*	MUCL 49453	Martinique	MK531811	MK531836	MK531825
*G. mirabile*	Cui 18236	Malaysia	**PZ630511**	**PZ661912**	**PZ661926**
*G. mirabile*	Cui 18237	Malaysia	MZ354960	MZ345731	MZ221674
*G. multiplicatum*	Dai 17395	Brazil	MZ354903	MZ345734	MZ221678
*G. multiplicatum*	Dai 12320	Yunnan, China	KU572490	–	KU572500
*G. multipileum*	Cui 13597	Hainan, China	MZ354899	MZ345732	MZ221675
*G. multipileum*	L4989	Yunnan, China	ON994249	OP508432	OP508447
*G. mutabile*	Dai 20414	Yunnan, China	MZ354977	MZ345735	MZ221680
*G. mutabile*	Yuan 2289^T^	Yunnan, China	JN383977	–	–
*G. obscuratum*	Lsh88^T^	Yunnan, China	ON994237	–	OP508450
*G. obscuratum*	Lsh89	Yunnan, China	ON994238	–	OP508451
*G. orbiforme*	Cui 13880	Hainan, China	MG279187	MG367523	MG367577
*G. orbiforme*	Cui 13891	Hainan, China	MZ354953	MZ345736	MZ221682
*G. oregonense*	JV 0108/93	United States	KF605620	MG367504	MG367558
*G. oregonense*	CBS 26588	United States	JQ781875	KJ143974	KJ143933
*G. ovisporum*	HKAS123193^T^	Guizhou, China	MZ519547	MZ547661	–
*G. ovisporum*	GACP20071602	Guizhou, China	MZ519548	MZ547662	–
*G. parvulum*	MUCL 52655	France	MK554770	MK554755	MK554717
*G. parvulum*	MUCL 47096	Cuba	MK554783	MK554742	MK554721
*G. philippii*	Cui 14443	Hainan, China	MG279188	MG367524	MG367578
*G. philippii*	Cui 14444	Hainan, China	MG279189	MG367525	MG367579
*G. phyllanthicola*	L4948^T^	Yunnan, China	PP869245	–	–
*G. phyllanthicola*	HL308	Yunnan, China	PP869246	–	–
*G. podocarpense*	JV 1504/126	Costa Rica	MZ354942	MZ345737	MZ221687
*G. podocarpense*	QCAM 6422	Ecuador	MF796661	–	–
*G. resinaceum*	LGAM 462	Greece	MG706250	MG837821	MG837858
*G. resinaceum*	LGAM 448	Greece	MG706249	MG837820	MG837857
*G. resinaceum*	MUCL 38956	Netherlands	MK554772	MK554747	MK554723
*G. sessile*	113FL	United States	MG654307	MG754867	MG754748
*G. sessile*	111TX	United States	MG654306	MG754866	MG754747
*G. shanxiense*	BJTC FM423^T^	Shanxi, China	MK764268	MK783940	MK783937
*G. shanxiense*	Dai 18921	Shanxi, China	MZ354909	MZ345740	MZ221691
*G. shennongii*	FHMU2290	Hainan, China	PP663109	–	–
*G. sichuanense*	Cui 16343	Guangxi, China	MZ354928	MZ345741	MZ221692
*G. sichuanense*	Dai 19651	Sri Lanka	MZ354929	MZ345742	MZ221693
*G. sinense*	Cui 14461	Guangxi, China	MZ354963	MZ345744	MZ221695
*G. sinense*	Wei 5327	Hainan, China	KF494998	MG367529	KF494976
*G. sinense*	Dai 20079	Guangxi, China	MZ354962	MZ345745	MZ221696
*G. suae*	L4651	Yunnan, China	PP869243	PP894784	PP894782
*G. suae*	L4817	Yunnan, China	PP869244	–	PP894783
*G. subangustisporum*	Cui 18597	Yunnan, China	MZ354980	MZ345746	MZ221700
*G. subangustisporum*	Cui 18592^T^	Yunnan, China	MZ354981	–	MZ221697
*G. subellipsoideum*	Cui 23398	Guangdong, China	**PZ630512**	–	**PZ661927**
*G. subellipsoideum*	Cui 23397	Guangdong, China	**PZ630513**	–	**PZ661928**
*G. subflexipes*	Cui 17247	Guangdong, China	MZ354921	MZ245395	MZ221645
*G. subflexipes*	Cui 17257	Guangdong, China	MZ354922	MZ245396	MZ221646
*G. sublobatum*	Cui 16804^T^	Australia	MZ354973	MZ345747	MZ221704
*G. sublobatum*	Cui 16806	Australia	**PZ630514**	–	**PZ661929**
*G. thailandicum*	HKAS104640	Thailand	MK848681	MK875831	MK875829
*G. thailandicum*	HKAS104641	Thailand	MK848682	MK875832	MK875830
*G. tongshanense*	Cui 17168^T^	Hubei, China	MZ354975	–	MZ221706
*G. tropicum*	Dai 16434	Hainan, China	MG279194	MG367532	MG367585
*G. tropicum*	Dai 19679	Guangdong, China	MZ354900	MZ358825	MZ221707
*G. tsugae*	Cui 14110	Jilin, China	MG279195	MG367533	MG367586
*G. tsugae*	Cui 14112	Jilin, China	MG279196	MG367534	MG367587
*G. weberianum*	Dai 19673	Guangdong, China	MZ354930	MZ358829	MZ221712
*G. weberianum*	Dai 19682	Guangdong, China	MZ354932	MZ358830	MZ221713
*G. williamsianum*	Dai 19611	Sri Lanka	MZ354949	MZ358832	MZ221717
*G. williamsianum*	Dai 16809	Thailand	MG279183	MG367535	MG367588
*G. williamsianum*	Dai 20553	Yunnan, China	MZ354948	MZ358831	MZ221716
*G. yunlingense*	Cui 16288^T^	Yunnan, China	MZ354915	–	MZ221718
*G. yunlingense*	Cui 17043	Yunnan, China	MZ354916	–	MZ221719
*G. yunnanense*	HL45^T^	Yunnan, China	ON994235	OP508422	OP508436
*G. yunnanense*	L4812	Yunnan, China	ON994236	OP508429	OP508443
*G. zonatum*	FL-03	United States	KJ143922	KJ143980	KJ143942
*G. zonatum*	FL-02	United States	KJ143921	KJ143979	KJ143941
*Haddowia macropora*	JV 1908/46^T^	France	MZ354870	MZ358847	MZ221720

Note: ^T^ in the table indicates the holotype specimen, and – refers to missing data. Sequences obtained in this study are in bold.

### Taxonomy

#### Ganoderma ficuum

Taxon classificationFungiPolyporalesPolyporaceae

B.K. Cui, Y.F. Sun, Y.M. Cheng & X.Y. Liu sp. nov.

E8EDFB9F-759F-5231-82C7-0E089F3B3605

864651

Figs 2, 3

##### Diagnosis.

Differs from other species in having sessile and imbricate basidiomata; pileal surface highly umbonate, with relatively coarse radial sulcations.

##### Etymology.

The epithet ‘*ficuum*’ refers to the host tree genus *Ficus*.

##### Holotype.

China • Guangdong Province, Shenzhen City, Futian District, on a living tree of *Ficus*, 12 Jun. 2024, Cui 23393.

##### Description.

***Basidiomata***: Annual, sessile. Pilei semicircular to shell-shaped, sometimes several overlapping and growing together, hard corky. Pileal surface highly arched upward, usually with concentric zones and coarse radial furrows at the center, moderately to strongly laccate, purplish brown to dark brown at maturity. Pileal margin usually acute, sometimes slightly involute, usually pale yellow. Pilei up to 9.5 cm long, 6 cm wide, and 1.6 cm thick. Pores circular to angular, 6–8 per mm, dissepiments slightly thick, entire. Pore surface white when juvenile, turning brown to dark brown when bruised, and buff to brown when dry. Context usually stratified, with the upper layer pale yellowish-brown and the lower layer brown, up to 1 cm thick. Tubes non-stratified, hard corky, brown to dark brown, up to 0.6 cm long.

***Hyphal structure***: Hyphal system trimitic; skeletal hyphae in the majority, yellowish brown, thick-walled to sub-solid, frequently dichotomously branched; generative hyphae with clamp connections, colorless, thin-walled, infrequent; binding hyphae pale yellow, thick-walled with narrow lumen, to sub-solid; all three types of hyphae IKI–, CB+; tissues darkening in KOH. Tube hyphae: skeletal hyphae 2.4–4.7 μm; generative hyphae 2.7–3.6 μm; binding hyphae 1.2–1.7 μm. Context hyphae: skeletal hyphae 2.7–5.8 μm; generative hyphae 1.4–1.7 μm; binding hyphae 1.1–1.9 μm.

***Spores***: Basidiospores ellipsoid, distinctly truncated after the apical beak is shed at maturity, pale yellowish brown; basidiospores double-walled, exospore wall smooth, endospore wall with moderately roughened punctate ornamentation, (6–)6.2–7.6(–8) × 4–5 (–5.3) μm, L = 7.23 μm, W = 4.5 μm, Q = 1.6 (n = 90/3).

##### Additional specimens.

China • Guangdong Province, Shenzhen City, Futian District, on a living *Ficus* tree, 11 Jun. 2024, Cui 23385, Cui 23386.

##### Note.

**Figure 1.**
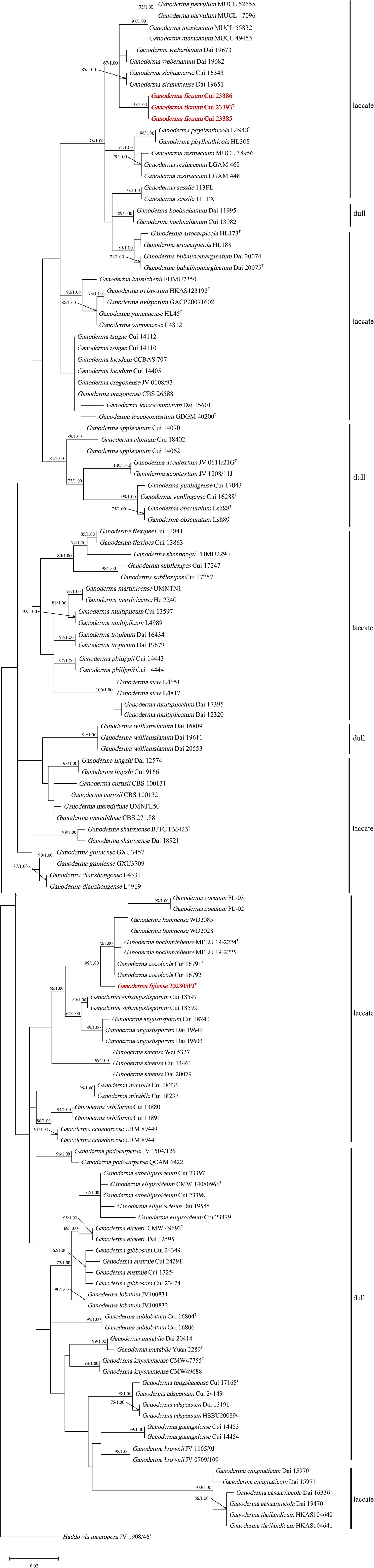
The phylogenetic tree of *Ganoderma* based on combined ITS+*TEF1-α*+*RPB2* sequence data. Bootstrap support values with a Maximum Likelihood (ML) of 50% or greater and Bayesian posterior probabilities (BPP) of 0.90 or greater are shown above the nodes as “ML-BS/BPP”. New species are indicated in bold red. Holotype specimens are noted using ^T^.

*Ganoderma ficuum* formed a sister clade with *G. weberianum*, *G. sichuanense*, *G. mexicanum* and *G. parvulum*, with relatively high bootstrap support (ML-BS = 67%, BPP = 1.00, Fig. 1). *Ganoderma ficuum* is morphologically distinct from *G. weberianum* in having a pileus that is strongly umbonate with relatively coarse radial sulcations, and a mature pileal surface ranging from purplish brown to dark brown, whereas *G. weberianum* is characterized by an applanate, flabelliform pileus with a mature surface colored purplish black to nearly black (Sun et al. 2022). *Ganoderma sichuanense* differs from *G. ficuum* by stipitate basidiomata (Wang et al. 2012). In contrast to the relatively applanate pileal surface of *G. mexicanum* and *G. parvulum*, that of *G. ficuum* is conspicuously arched upward. Furthermore, the basidiospores of *G. ficuum* are 6.2–7.6 × 4–5 μm, which are significantly smaller than those of *G. mexicanum* (8–9 × 6–7 μm) and *G. parvulum* (8–9.5 × 5.5–6.5 μm) (Cabarroi-Hernández et al. 2019).

**Figure 2.**
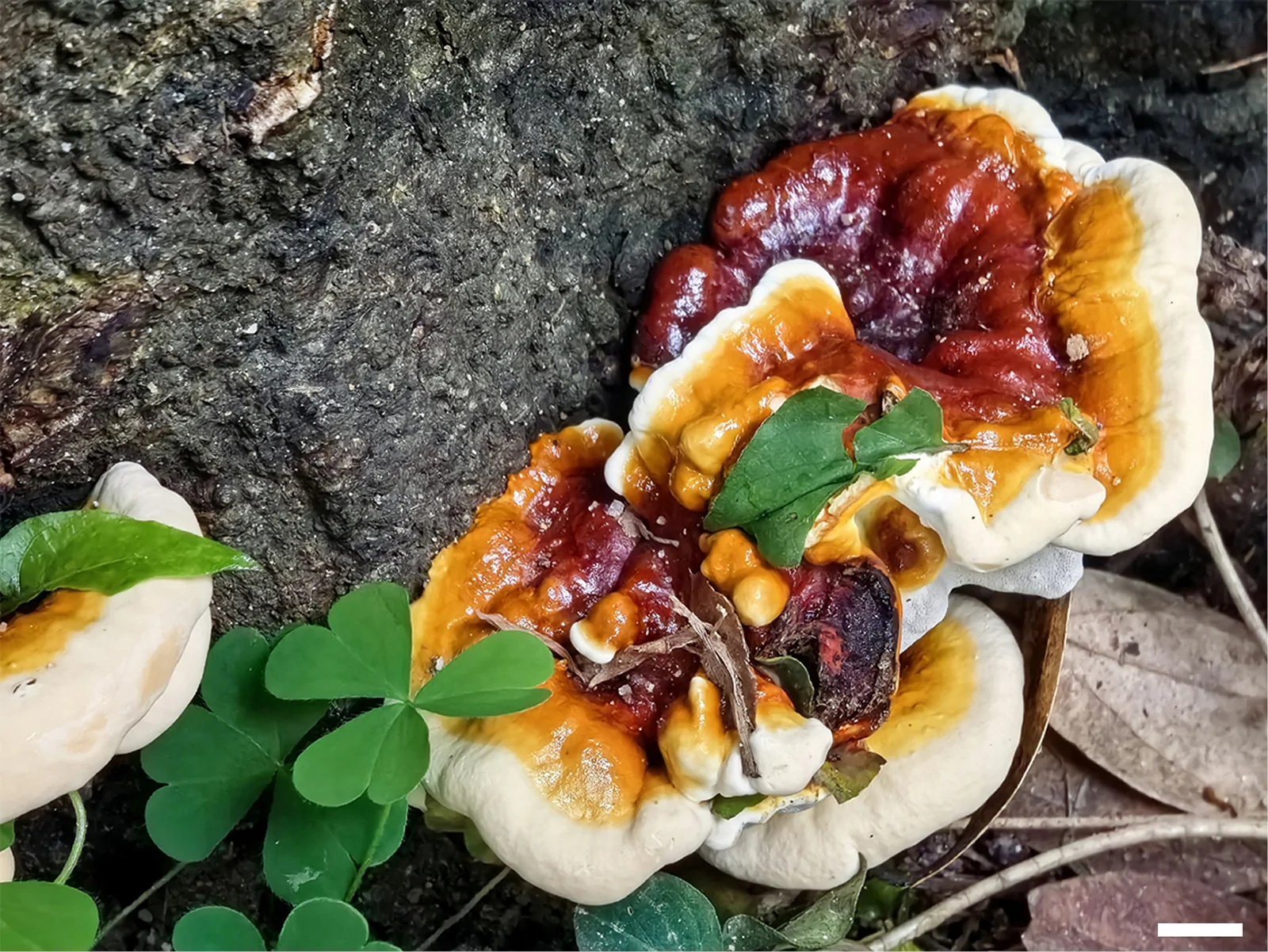
Basidiomata of *Ganoderma ficuum*. Scale bar: 1 cm.

**Figure 3.**
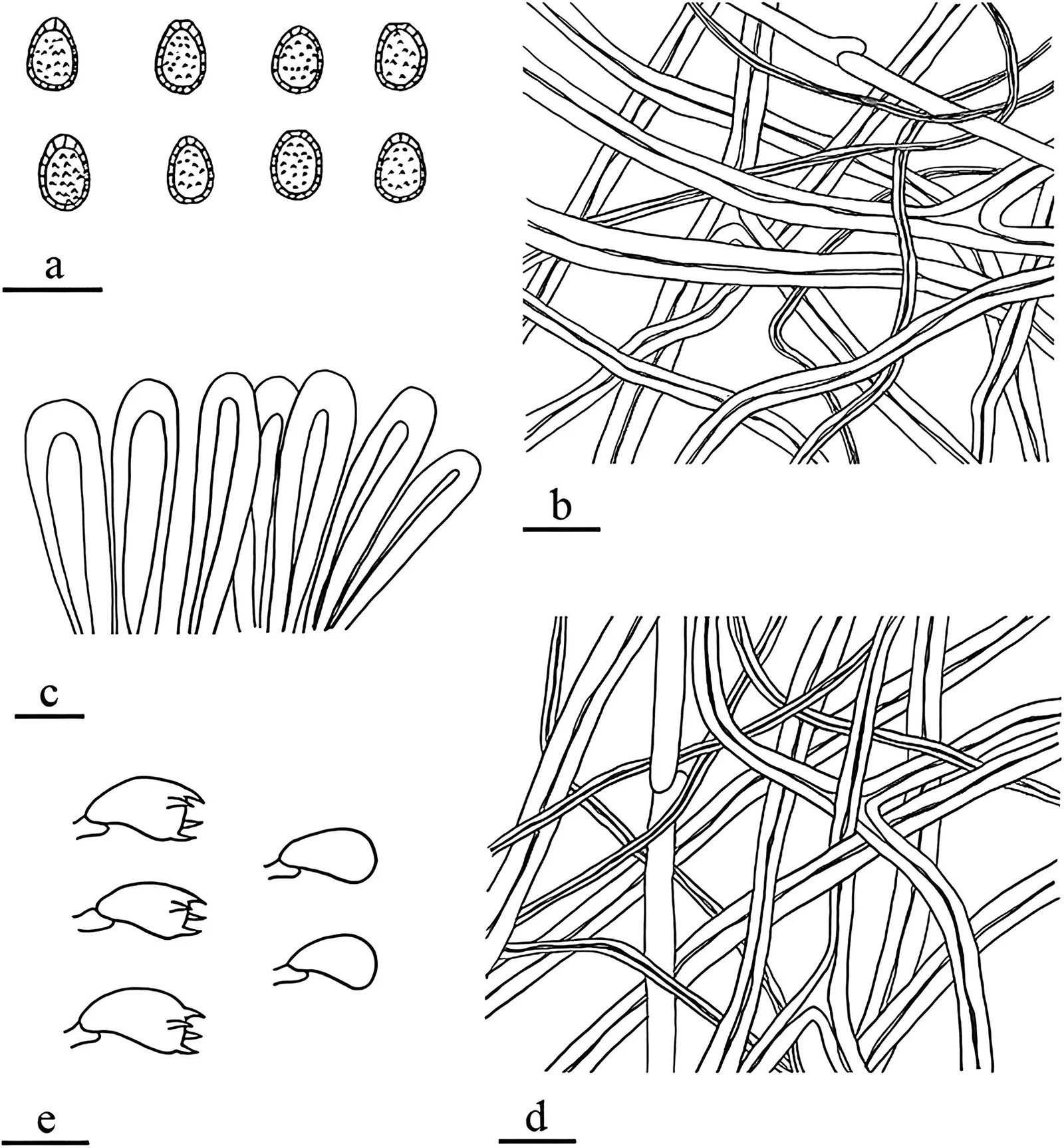
Microscopic structures of *Ganoderma ficuum* (drawn from Cui 23393). **a**. Basidiospores; **b**. Hyphae from trama; **c**. Apical cells from the cuticle; **d**. Hyphae from context; **e**. Basidia and basidioles. Scale bars: 10 μm.

#### Ganoderma fijiense

Taxon classificationFungiPolyporalesPolyporaceae

B.K. Cui, Y.F. Sun & X.Y. Liu sp. nov.

FDDEF705-77E5-5D86-B6B1-F44C8A8B8D93

864652

Figs 4, 5

##### Diagnosis.

Differs from other species by sessile basidiomata with reddish brown to dark brown pileal surface, and basidiospores narrowly ellipsoid, with the endospore wall heavily roughened with punctate ornamentation.

##### Etymology.

The epithet ‘*fijiense*’ refers to the type locality, Fiji.

##### Holotype.

Fiji • Nadi, on the trunk of a *Cocos* tree, collected by Xingsheng Lin and Zhixin Huang, May 2023, 202305FJ.

##### Description.

***Basidiomata***: Annual, sessile or with a constricted base. Pilei reniform to orbicular, hard corky. Pileal surface usually with concentric zones, more obvious near the margin, and usually with fine or broad (radial) furrows at the center, slightly to moderately laccate when juvenile, reddish brown to dark brown. Pileal margin usually obtuse, pale yellow when juvenile, becoming yellowish brown or reddish brown at maturity. Pilei up to 5.6 cm long, 5.2 cm wide, and 2.3 cm thick. Pores circular, 5–7 per mm, dissepiments thick, mostly entire. Pore surface white when juvenile, turning brown to dark brown when bruised, and buff to pale brown when dry. Context usually non-stratified, with black melanoid lines, hard corky, dark brown, up to 1 cm thick. Tubes non-stratified, hard corky, pale brown to brown, up to 1.3 cm long.

**Figure 4.**
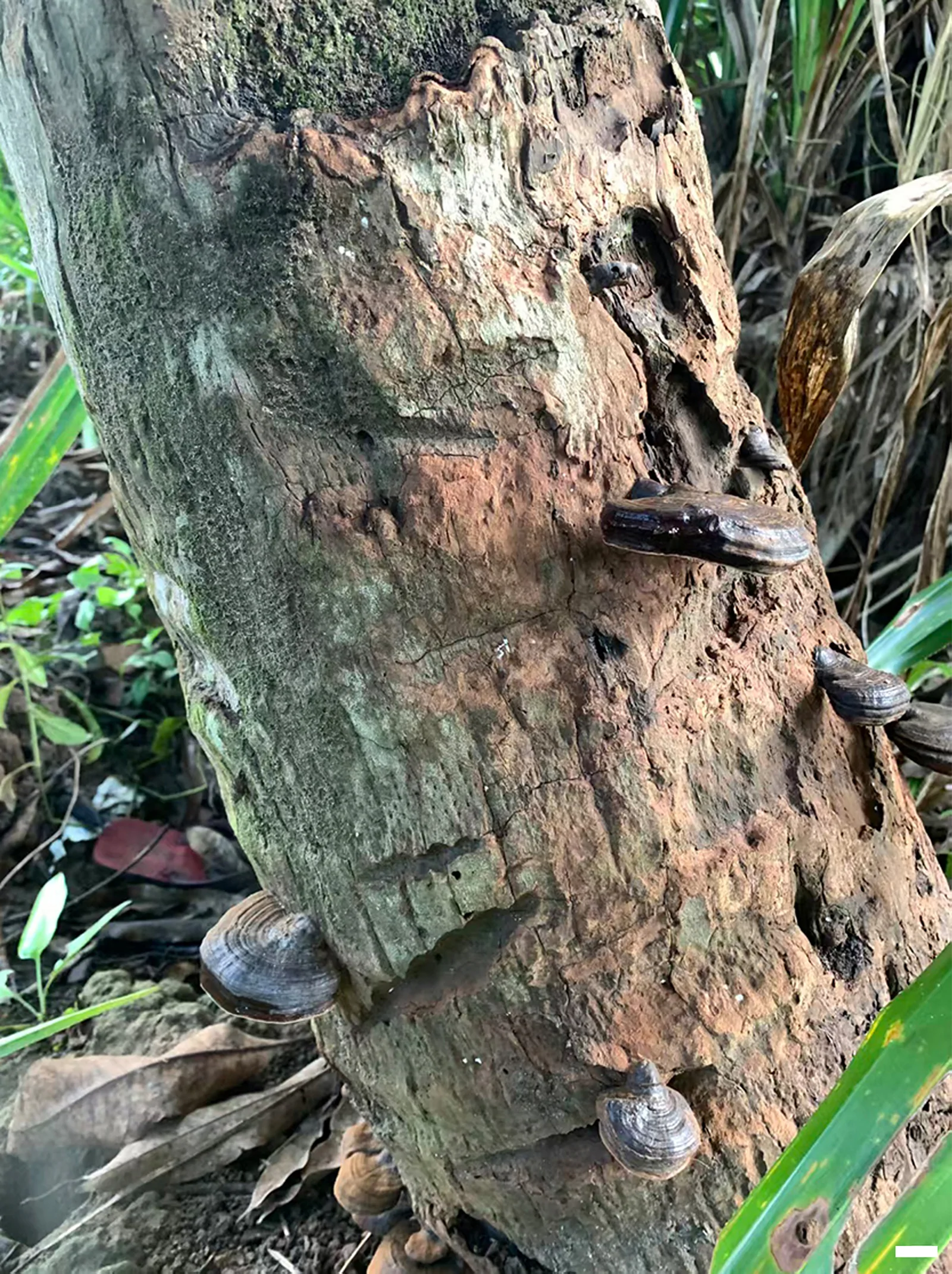
Basidiomata of *Ganoderma fijiense*. Scale bar: 1 cm.

***Hyphal structure***: Hyphal system trimitic; skeletal hyphae in the majority, yellowish brown, thick-walled with narrow lumen or sub-solid, frequently dichotomously branched; generative hyphae with clamp connections, colorless, thin-walled, infrequent; binding hyphae pale yellow, thick-walled with narrow lumen to sub-solid; all hyphae IKI–, CB+; tissues darkening in KOH. Context: skeletal hyphae 1.7–4 μm, generative hyphae 2.2–3 μm, binding hyphae 1–1.6 μm. Tube: skeletal hyphae 1.5–3.8 μm, generative hyphae 2–2.9 μm, binding hyphae 0.9–2 μm.

**Figure 5.**
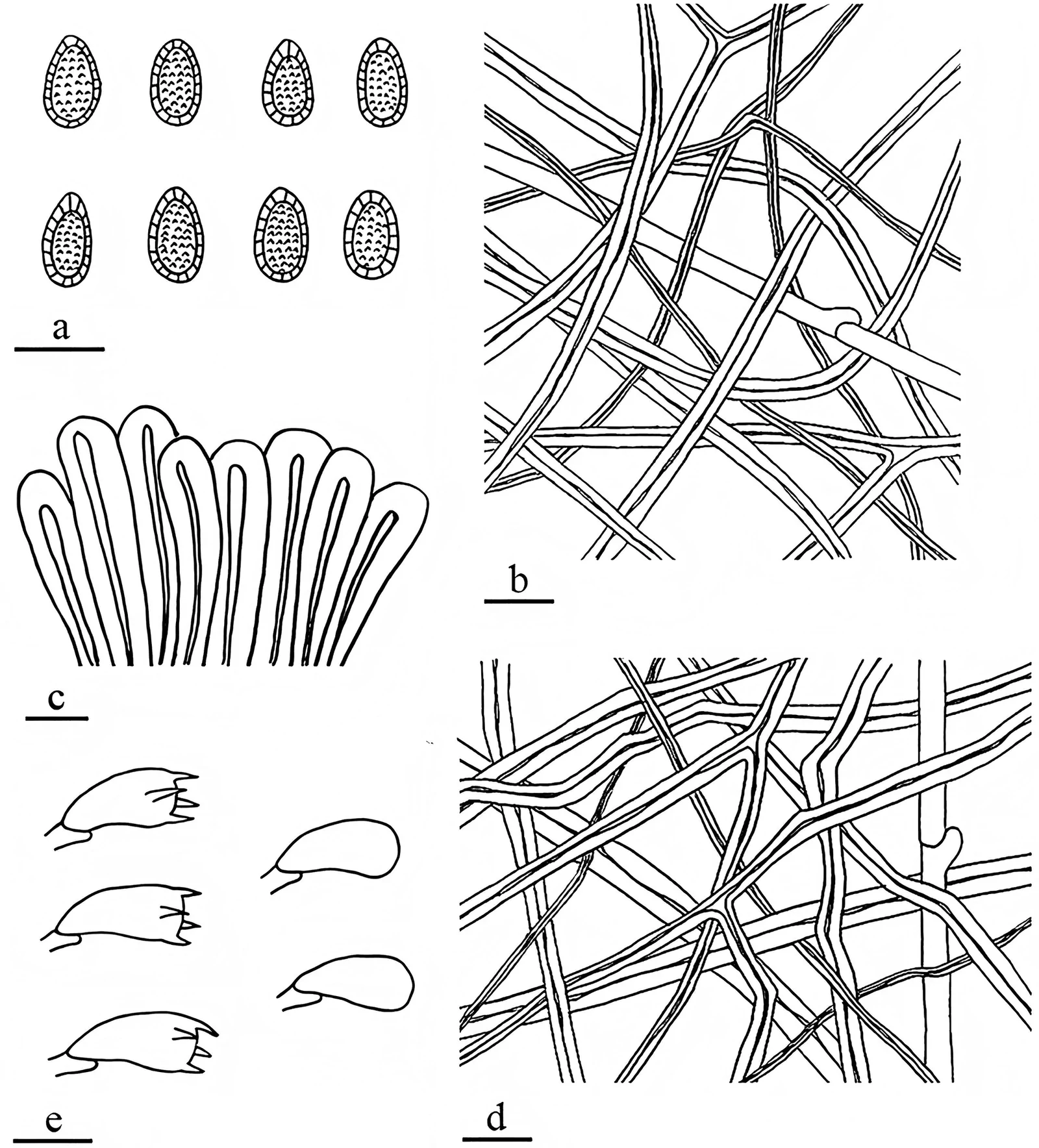
Microscopic structures of *Ganoderma fijiense* (drawn from 202305FJ). **a**. Basidiospores; **b**. Hyphae from trama; **c**. Apical cells from the cuticle; **d**. Hyphae from context; **e**. Basidia and basidioles. Scale bars: 10 μm.

***Spores***: Basidiospores narrowly ellipsoid, with an apiculus and truncated, pale yellowish brown, IKI–, CB+, double-walled, exospore wall smooth, endospore wall with heavily roughened punctate ornamentation, (8–)8.2–11(–11.4) × (4.3–)4.7–5.4(–5.8) μm, L = 10.09 μm, W = 4.96 μm, Q = 2 (n = 60/1).

##### Note.

*Ganoderma fijiense* formed a sister clade comprising *G. zonatum*, *G. cocoicola*, *G. hochiminhense* and *G. boninense*, with high support (ML-BS = 99%, BPP = 1.00, Fig. 1). The species *Ganoderma hochiminhense* grows on *Areca* trees, while *G. fijiense* grow on *Cocos* trees. Both *G. fijiense* and *G. cocoicola* may occur in the same host and possess laccate pileal surfaces. However, *G. fijiense* can be distinguished from *G. cocoicola* by its context, which usually contains black melanoid lines, a feature absent in the latter. Additionally, *G. fijiense* has more densely packed pores, measuring 5–7 per millimeter, whereas *G. cocoicola* has relatively sparser pores, at 4–6 per millimeter (Sun et al. 2022). Among them, the basidiospores of *G. zonatum* are significantly larger (reaching up to 11–14 µm in length), far exceeding those of *G. fijiense* (8.2–11.1 µm). The spore size of *G. boninense* (ca. 10 × 7 µm) is wider than that of *G. fijiense* (4.7–5.4 µm), and more ovoid in shape, which can effectively distinguish it from *G. fijiense* (Gottlieb and Wright 1999; Seo and Kirk 2000).

## Discussion

As the most species-rich genus in *Ganodermataceae*, *Ganoderma* has been extensively studied by numerous scholars (He et al. 2022). With the deepening of research on *Ganoderma*, an increasing number of new species were being discovered and described. Based on morphological characteristics and multi-gene phylogenetic analyses, this study reports two new species from China and Fiji: *Ganoderma ficuum* and *G. fijiense*. Both fungi conform to the taxonomic definition of this genus: the pileal surface is laccate with various decorative ornamentations, their basidiospores are ellipsoid or ovoid, double-walled with thick walls, and the endospore wall has distinct verrucose ornamentations. Comparison revealed that *G. ficuum* and *G. fijiense* occupy distinct, distantly separated positions in the multilocus phylogenetic tree, and their basidiomata exhibit significant differences in both macro- and microscopical characters. For instance, *G. fijiense* differs from *G. ficuum* by imbricate basidiomata, and narrowly ellipsoid basidiospores.

Guangdong Province, located south of the Nanling Mountains, falls within a typical tropical and subtropical monsoon climate zone. In addition to *Ganoderma ficuum*, *G. casuarinicola* J.H. Xing et al., *G. subflexipes* B.K. Cui et al., *G. weberianum* and at least 10 other species have been discovered in Guangdong Province (Xing et al. 2018; Luangharn et al. 2019; He et al. 2024). The phylogenetic tree shows that *G. ficuum* is closely related to *G. weberianum*, *G. sichuanense*, *G. mexicanum* and *G. parvulum*. *Ganoderma ficuum*, *G. sichuanense* and *G. weberianum* are typically associated with decaying wood of broadleaf trees. In contrast, the host records of *G. mexicanum* and *G. parvulum* tend to be more frequently associated with palm (*Arecaceae*) or other monocotyledonous plant remains (Cabarroi-Hernández et al. 2019). This divergence in host preference suggests that different species may have adopted distinct adaptive strategies for lignin degradation or for microhabitat conditions. From a geographical perspective, *G. sichuanense* and *G. ficuum* are restricted to warm-temperate to tropical regions of East Asia, while *G. mexicanum* and *G. parvulum* are distributed in the neotropics and are members of the *G. weberianum-resinaceum* lineage (Cabarroi-Hernández et al. 2019). This disjunct, transcontinental distribution pattern may be related to dispersal events via land bridges or long-distance spore dispersal during geological periods (Moncalvo and Buchanan 2008) and warrants further verification through more extensive population genetic analyses in the future.

According to the phylogenetic tree constructed from combined multi-gene sequences, *G. fijiense* is closely related to *G. zonatum*, *G. cocoicola*, *G. hochiminhense* and *G. boninense*. The species *Ganoderma hochiminhense* is distributed in mainland Southeast Asia (Vietnam, Thailand), with the type specimen collected from a stump of *Areca* spp. in Vietnam; subsequent collections were made in Thailand from dead oil palm (*Elaeis guineensis*) trunks (Luangharn et al. 2021). *Ganoderma cocoicola* is known to be distributed in Australia and Malaysia (Sun et al. 2022), whereas *G. fijiense* has been recorded only in Fiji to date. Although they share a common ecological preference, with hosts all belonging to *Arecaceae* (palms), they are clearly geographically isolated from one another. This distribution pattern suggests that geographical isolation among the new species with related taxa may have played an important role in the diversification of this group.

Although this study has described two new species, some issues still require further investigation. On the one hand, currently only a few gene fragments such as ITS and *TEF1-α* have been relied upon; future whole-genome analyses should be conducted to reveal the stability of their phylogenetic relationships. On the other hand, current records of *Ganoderma* in Pacific Island Countries are limited, and future efforts should expand field surveys across adjacent islands and continental areas, combined with environmental factors, to assess their niche differentiation.

## Supplementary Material

XML Treatment for Ganoderma ficuum

XML Treatment for Ganoderma fijiense

## Additional information

### Artificial Intelligence (AI) use

The authors accept full responsibility for the content of the manuscript, including the disclosure of any use of AI.

No AI tools were used in the preparation of this manuscript.

### Author ORCIDs

X.-Y. Liu https://orcid.org/0009-0004-7151-3230

Y.-L. Liu https://orcid.org/0000-0001-8682-6599

Z.-X. Lin https://orcid.org/0000-0003-1697-7919

D.-M. Lin https://orcid.org/0009-0004-1173-5173

X.-S. Lin https://orcid.org/0009-0005-9051-8819

Y.-M. Cheng https://orcid.org/0009-0000-8056-3441

Y.-F. Sun https://orcid.org/0000-0003-3997-3662

B.-K. Cui https://orcid.org/0000-0003-3059-9344

### Data availability

All of the data that support the findings of this study are available in the main text.
